# Recent Trends in Non-Destructive Testing Approaches for Composite Materials: A Review of Successful Implementations

**DOI:** 10.3390/ma18133146

**Published:** 2025-07-02

**Authors:** Jan Lean Tai, Mohamed Thariq Hameed Sultan, Andrzej Łukaszewicz, Jerzy Józwik, Zbigniew Oksiuta, Farah Syazwani Shahar

**Affiliations:** 1 Department of Aerospace Engineering, Faculty of Engineering, Universiti Putra Malaysia, Serdang 43400, Selangor, Malaysia; gs63398@student.upm.edu.my (J.L.T.); farahsyazwani@upm.edu.my (F.S.S.); 2 Laboratory of Biocomposite Technology, Institute of Tropical Forestry and Forest Products (INTROP), Universiti Putra Malaysia, Serdang 43400, Selangor, Malaysia; 3 Aerospace Malaysia Innovation Centre [944751-A], Prime Minister’s Department, MIGHT Partnership Hub, Jalan Impact, Cyberjaya 63600, Selangor, Malaysia; 4 Institute of Mechanical Engineering, Faculty of Mechanical Engineering, Bialystok University of Technology, Wiejska St. 45C, 15-351 Bialystok, Poland; 5 Department of Production Engineering, Faculty of Mechanical Engineering, Lublin University of Technology, Nadbystrzycka St. 36, 20-618 Lublin, Poland; j.jozwik@pollub.pl; 6 Institute of Technical Sciences and Aviation, The State University of Applied Sciences in Chełm, Pocztowa St. 54, 22-100 Chełm, Poland; 7 Institute of Biomedical Engineering, Faculty of Mechanical Engineering, Bialystok University of Technology, Wiejska St. 45C, 15-351 Bialystok, Poland; z.oksiuta@pb.edu.pl

**Keywords:** composite materials, non-destructive testing (NDT), ultrasonic testing (UT), infrared thermography testing (IRT), X-ray computed tomography (XCT), multi-sensors

## Abstract

Non-destructive testing (NDT) methods are critical for evaluating the structural integrity of and detecting defects in composite materials across industries such as aerospace and renewable energy. This review examines the recent trends and successful implementations of NDT approaches for composite materials, focusing on articles published between 2015 and 2025. A systematic literature review identified 120 relevant articles, highlighting techniques such as ultrasonic testing (UT), acoustic emission testing (AET), thermography (TR), radiographic testing (RT), eddy current testing (ECT), infrared thermography (IRT), X-ray computed tomography (XCT), and digital radiography testing (DRT). These methods effectively detect defects such as debonding, delamination, and voids in fiber-reinforced polymer (FRP) composites. The selection of NDT approaches depends on the material properties, defect types, and testing conditions. Although each technique has advantages and limitations, combining multiple NDT methods enhances the quality assessment of composite materials. This review provides insights into the capabilities and limitations of various NDT techniques and suggests future research directions for combining NDT methods to improve quality control in composite material manufacturing. Future trends include adopting multimodal NDT systems, integrating digital twin and Industry 4.0 technologies, utilizing embedded and wireless structural health monitoring, and applying artificial intelligence for automated defect interpretation. These advancements are promising for transforming NDT into an intelligent, predictive, and integrated quality assurance system.

## 1. Introduction

Composite materials have become revolutionary in various industries and offer numerous advantages over traditional metallic materials. Although the initial costs of composite materials may be higher, their benefits, such as superior strength-to-weight ratios, make them attractive options [1]. This characteristic is particularly valuable in the era of rising raw material transportation costs because the lightweight nature of the composite materials enhances transportation efficiency and reduces overall expenses.

Composite pipes, for example, possess excellent insulating properties, making them suitable for environments in which metallic pipes are impractical owing to electrical conductivity issues. This versatility broadens the range of applications of the composite pipes. Additionally, composite pipes demonstrate exceptional corrosion resistance compared to metallic pipes, enhancing their durability and significantly reducing maintenance costs over their lifespan [2]. Consequently, the long-term economic benefits of composite pipes have increased their popularity in various industries and applications.

The growing demand for composite pipes has resulted in a proliferation of manufacturers, each offering unique combinations and manufacturing processes tailored to specific needs. However, this diversity presents a challenge in quality control, as it ensures the reliability and functionality of composite pipes, particularly in critical applications, such as process plants and infrastructure.

Hydrostatic testing is a commonly used quality control method for the shipment of composite pipes. Hydrostatic testing is preferred over other methods because it ensures that composite pipes meet the required specifications for pressure resistance and safety. Although effective in detecting defects, this method is resource-intensive and time-consuming. Identifying defective composite pipes at this stage can result in a substantial waste of labor and resources.

To address the challenges of ensuring composite pipe integrity, manufacturers and industry stakeholders are exploring advancements in non-destructive testing (NDT) methods and inspection techniques. NDT methods enable defect detection without damaging pipes and significantly reduce the risk of wasted resources. NDT encompasses various testing methods and has proven to be effective across multiple fields, including applications in composite materials.

This review aims to provide a comprehensive synthesis of current state-of-the-art NDT methods for composite materials, identify prevailing research trends, and propose future perspectives on the integration of artificial intelligence (AI), digital twins, and multimodal inspection strategies. This study highlights the strengths, limitations, and emerging opportunities of various NDT techniques by analyzing 120 peer-reviewed articles published between 2015 and 2025 that met our inclusion criteria.

## 2. Prevailing Trend in the Application of NDT Methods to Composite Materials

In the field of composite materials, non-destructive evaluation (NDE) techniques have become increasingly important for assessing structural integrity and detecting potential defects without compromising the functionality of the material.

The reliability and structural integrity of fiber-reinforced polymer (FRP) composite materials are of paramount importance, particularly in critical applications in aerospace [3], infrastructure, automotive, and renewable energy industries. Researchers have made significant efforts to develop and refine NDT methods/techniques for evaluating damage and defects in advanced materials. Gandhi et al. [4] emphasize the need to consider factors such as the material properties, defect types, and testing conditions when selecting appropriate NDT approaches for composite structures.

A key article reviewed by Gholizadeh [5] provides a comprehensive overview of the various NDT methods available for damage detection in FRP composites. The authors highlighted the use of ultrasonic testing (UT), acoustic emission testing (AET), and thermography (TR) as effective techniques for identifying defects and monitoring the structural health of composite structures. These non-destructive approaches are crucial for ensuring the reliable performance of composite components without the need for destructive testing.

Herrmann et al. [6] presented a comprehensive study on the application of various NDT methods, such as UT, TR, and radiographic testing (RT) techniques, with insights into the capabilities and limitations of these methods in terms of defect detection, characterization, and quantification in composite materials. Sathawane and Singh [7] investigated the specific applications of NDT methods in the aerospace industry. Researchers have focused on the use of UT, eddy current testing (ECT), and infrared thermography testing (IRT) to evaluate the composite materials used in aircraft structures.

X-ray computed tomography (XCT) is an emerging NDT technique for composite materials. Elkolali et al. [8] utilized XCT to determine the void content in FRP composites, which is a critical parameter affecting the mechanical performance of these materials. Digital radiography testing (DRT) and XCT have been successfully used to detect debonding, delamination, and isolated and grouped voids in carbon fiber-reinforced polymer (CFRP) specimens. Compared to both NDT techniques, DRT offers the advantage of being relatively low-cost [9]. However, it is worth noting that the size of the XCT machine can limit the size of the test specimen that can be used for examination.

Chen et al. [10] provided a comprehensive review of various NDT and evaluation techniques for detecting defects in FRP composites, with a focus on UT methods. The authors discussed the principles, advantages, and limitations of techniques such as pulse-echo, through-transmission, and phased-array ultrasonic testing (PAUT), as well as their applications in the assessment of composite materials.

The UT and PAUT of FRP materials present unique challenges owing to the anisotropic nature of the FRP composites. This anisotropy affects the propagation of ultrasonic waves through a material. The speed of sound, attenuation, and reflection characteristics can differ significantly depending on the direction of the fibers and the loss of ultrasound energy [11], particularly when dealing with thick sections of composite materials [12]. This further creates the challenge of developing an appropriate calibration procedure for a reference calibration block of an anisotropic nature.

Despite these challenges, UT and PAUT remain valuable methods for assessing the integrity of FRP materials, and their effectiveness has been proven by several researchers [13,14].

Radiofrequency (RF) testing is another prominent NDT technique. Chaki and Krawczak [15] highlighted the non-destructive health monitoring of structural composites, underscoring the potential of RF-based methods, such as ECT, microwave (MW), and RF-based thermography. These techniques leverage the anisotropic electrical properties of CFRP composites to detect defects [16].

Li and Meng [17] categorize RF-based NDT methods for CFRP composites. They categorized these techniques into four types: electromagnetic induction, resonance, RF-based thermography, and RF wave propagation. These methods demonstrate the ability to detect various types of damage, including impact damage [17].

Suhasini and Reddy [18] provided a comprehensive overview of the different types of defects encountered in composite materials and the parameters that influence their detection and characterization using NDT methods. This study encompasses various NDT techniques, including visual inspection, UT, and RT. Zhang et al. [19] focused on the detection of tiny defects in organic insulating materials, including composite materials. This review discusses the use of techniques, such as AET, IRT, and UT, to identify microdefects and their potential applications in the composite industry.

The extensive utilization of NDT techniques for composite materials is evident. The following subsection provides a concise overview of various NDT methods employed by researchers.

### 2.1. Ultrasonic-Based Testing Methods Testing

Ultrasonic-based techniques, including conventional UT and advanced PAUT, are widely used for defect detection in composite materials owing to their high penetration capability and resolution. The following subsections explore both techniques in detail.

#### 2.1.1. Ultrasonic Testing (UT)

UT has long been the preferred choice for the NDT of metal parts and assemblies owing to its numerous advantages. The ability of ultrasonic waves to effectively penetrate and propagate through metallic materials makes them an ideal tool for the detection and characterization of internal defects, such as cracks, voids, inclusions, corrosion, and material discontinuities, which can compromise the structural integrity of critical industrial components [20,21,22].

In metal fabrication and manufacturing industries, UT is extensively utilized to ensure the quality and reliability of welds, castings, forgings, and other metal products [23,24]. This technique allows for the rapid and cost-effective inspection of these components, enabling manufacturers to identify and address potential flaws before escalating into larger, more serious problems.

Similarly, in the oil and gas sector, UT is widely employed for inspecting pipelines, storage tanks, pressure vessels, and other metal infrastructure [25,26]. The ability of UT to operate through coatings and various surface conditions makes them a versatile option for the in-service monitoring and assessment of these critical assets, helping to prevent catastrophic failures and ensure the safe operation of industrial facilities [27,28].

Beyond the metal industry, UT also find applications in the inspection of concrete structures [29], composite materials, and biological tissues, demonstrating their adaptability and broad utility across diverse industries and applications. However, UT applications are not limited to metals. It has also emerged as a powerful technique for the detection and characterization of various defects and anomalies in composite structures.

One of the primary considerations in the application of UT for composite materials is the appropriate selection of the operating frequency [30,31,32]. The choice of frequency is a trade-off between the spatial resolution and depth of penetration [33]. Higher frequencies, typically in the range of 10–25 MHz, offer better resolution for the detection of small-scale defects, such as delamination, fiber waviness, and microcracks near the surface of the composite. However, these higher frequencies tend to have limited penetration depths, which is a concern for thicker and multilayered composite structures.

Conversely, lower frequencies, typically in the range of 1–5 MHz, are better suited for inspecting deeper bulk defects in composite laminates. However, this may compromise their ability to resolve these small-scale flaws. Optimizing the frequency selection is crucial to ensure the effective detection and characterization of the full range of potential defects in composite materials.

In addition to the choice of frequency, the anisotropic nature of composite materials presents a challenge for UT [34]. The varying acoustic properties of the fiber and matrix materials, as well as the directionality of the fibers, can lead to complex wave propagation and attenuation patterns [35]. This can result in the need for advanced signal processing and analysis techniques to accurately interpret the ultrasonic data and extract relevant information regarding the structural integrity of the composite.

Several studies have focused on improving the effectiveness of UT in composite materials. For instance, Matalgah et al. [36] focused on the automated quantification of interlaminar delamination in composite materials using UT techniques. Ellison and Kim [37] focused on the estimation of shadowed delamination areas in composite materials using UT C-scans. This study addresses methods for estimating the true size of the delamination, even when they are partially obscured, using UT techniques. Ma et al. [38] developed an approach that combines advanced signal processing and analysis to accurately identify and characterize the depth and extent of delamination defects within the composite laminate structure.

UT has also been applied to other composite materials beyond CFRP. Jasiūnienė et al. [39] focused on the application of UT for the evaluation of complex titanium structures, which are increasingly used in aerospace and other industries. This study highlights the versatility of UT in addressing the challenges associated with the inspection of intricate and anisotropic materials, which are also relevant to the analysis of composite structures. Ibrahim [40] investigated the use of UT to inspect polymer matrix composites. Hybrid composites, which combine different types of reinforcements such as carbon and glass fibers, are becoming increasingly prevalent in various industries. The UT-based inspection of these materials could provide valuable information about their structural properties and the presence of defects.

Many researchers have combined and compared different NDT methods to improve the sensitivity and resolution of UT. Rizwan et al. [41] investigated the use of pulse-echo and PAUT for the imaging of thick CFRP components. The combination of pulse-echo and PAUT techniques can potentially improve the sensitivity and resolution of UT for highly attenuating and scattering materials, such as thick CFRP laminates. Kappatos et al. [42] compared different UT techniques, such as pulse-echo, pitch-catch, and PAUT for the detection of defects in composite components. Seo et al. [43] presented a novel technique for the detection of debonding in composite structures using a temporary attachment method and various NDT approaches, including impact-based methods and UT.

Teng and Zhou [44] introduced a novel NDT method for CFRP composites that combines ultrasonic imaging with TR analysis. By integrating these complementary techniques, researchers have demonstrated the ability to detect and characterize a wider range of defects, including delamination, impact damage, and porosity, with improved accuracy and reliability compared with traditional UT or TR methods alone.

Evans et al. [45] investigated the comparison of XCT and UT techniques for the evaluation of composite materials. XCT is a widely used non-destructive imaging technique that can provide detailed three-dimensional (3D) information about the internal structure of composite components.

Similarly, Amif and Jack [46] presented a novel method for the non-destructive extraction of the 3D geometry of an individual lamina within a CFRP laminate, with a specific focus on the characterization of subsurface wrinkles. Santos et al. [47] provided a comprehensive review of the use of ultrasonic C-scan techniques for the assessment of damage in composite materials. The advantages of C-scanning over other methods include the detection and characterization of various defects, including delamination, impact damage, and fiber waviness. The C-scan shows the top view of the test object for better defect identification [48,49].

In an evaluation of large or complex composite structures, Lim et al. [50] explored the use of a multijoint robotic system for the automated UT of CFRP components. Researchers have demonstrated that the integration of robotic technology can enhance the accuracy, repeatability, and efficiency of UT-based inspection [51,52,53].

#### 2.1.2. Ultrasonic Phased Array Ultrasonic Testing (PAUT)

The PAUT is an advanced UT technique employed to inspect and assess the integrity of various materials and structures. PAUT utilizes an array of ultrasonic transducers that can be individually controlled to adjust the beam angle, focus, and shape, offering more precise and flexible inspections than conventional UT methods [54]. This sophisticated technique is extensively used in industries such as oil and gas, power generation, and aerospace for detecting and characterizing defects, including corrosion, cracks, and weld imperfections, with high accuracy and efficiency [55].

Antin et al. [56] compared PAUT with other NDT methods for inspecting CFRP ropes and found that establishing good coupling between the PAUT probe and rope surface was challenging due to the presence of a protective coating. Camineroa et al. [57] assessed the capability of PAUT to locate and characterize artificial inclusions of different shapes, sizes, and materials embedded in CFRP laminates. The results indicated that the PAUT could accurately locate most of the inclusions; however, determining their shape and size was challenging owing to the high signal attenuation and distortion of the CFRP composite.

Conventional UT has limitations when inspecting composites owing to their anisotropic structure, high attenuation, and low signal-to-noise ratio. PAUT can overcome these limitations by providing the capability to focus and steer ultrasonic signals. Taheri et al. [14] evaluated the capability of PAUT versus UT to detect flaws in GFRP composite samples. The results showed that the PAUT could detect flaws as small as 0.8 mm with a penetration depth of up to 25 mm and that the PAUT signals had better characteristics than the UT signals.

Thermoplastic composite pipes (TCPs) have emerged as a promising alternative to traditional metal pipes, offering superior corrosion resistance, fatigue life, and lower installation and maintenance costs. However, as TCP structures become thicker and more complex, inspecting them using traditional UT techniques has become increasingly challenging. Conventional UT methods, designed primarily for homogeneous and isotropic materials, such as metals, struggle to effectively detect defects in thick multilayered composite structures. Mohd Tahir and Echtermeyer [58] explore the feasibility of using PAUT combined with a classical time-corrected gain (TCG) method to inspect thick, glass fiber-reinforced TCP and demonstrate its effectiveness in detecting various defects, such as artificial flat-bottom holes (FBHs) and a deliberately fabricated defect within the composite laminate layers. FBHs are widely recognized as standard reference features for ultrasonic calibration because of their consistent geometry and well-defined signal-response characteristics [59].

**Figure 1:**
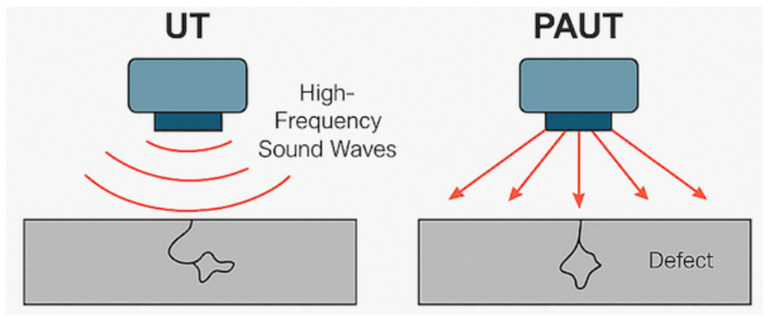
Schematic of UT and PAUT operating principles.

Table 1 lists the key features of the ultrasonic-based testing methods, including conventional UT and PAUT. It provides a comparative summary of their operational descriptions, advantages, limitations, and typical industrial applications for composite-material inspection. Figure 1 illustrates the operating principles of the conventional UT and PAUT. In UT, a single transducer emits high-frequency sound waves that reflect off internal defects, whereas in PAUT, multiple elements in a phased array probe allow for beam steering and dynamic focusing, enabling more comprehensive flaw detection in complex composite structures.

### 2.2. Electromagnetic-Based Testing Methods

Electromagnetic-based NDT techniques are highly effective for inspecting conductive composite materials, especially CFRP. These methods operate by inducing and analyzing eddy currents within a material, allowing for the detection of surface and near-surface defects. The most commonly used technique is the conventional ECT, which provides a rapid and sensitive inspection without requiring surface contact. Recent advancements have led to the development of pulse eddy current testing (PECT), which is a time–domain method that enables deeper penetration and improved signal interpretation. The following subsections describe these two methods in detail.

#### 2.2.1. Eddy Current Testing (ECT)

ECT is a versatile and highly sensitive NDE technique that relies on electromagnetic induction principles to detect and characterize defects or material properties in conductive materials [60]. By introducing an alternating current into the probe coil, ECT generates a magnetic field that induces eddy currents within the conductive material being inspected. These eddy currents, in turn, create a magnetic field that can be measured and analyzed to reveal information about the properties of the material and the presence of defects or anomalies.

ECT is widely used in various industries, including aerospace, automotive, and infrastructure, owing to its ability to quickly and accurately inspect components and ensure their structural integrity [61]. This technique is particularly useful for detecting surface and near-surface defects, such as cracks and changes in the material composition. Additionally, ECT can be used to measure material properties, such as conductivity and permeability, making it a valuable tool for material characterization and quality control [60].

One of the key advantages of ECT is its high sensitivity to small defects and variations in the material properties [62]. This sensitivity allows for the early detection of potential issues, enabling timely repair or replacement, and ultimately preventing catastrophic failures. Moreover, ECT can be performed without direct contact with the inspected material, making it suitable for use in situations where access is limited or surface conditions are challenging.

Several types of ECT probes have been designed for specific applications, offering unique benefits. For example, absolute probes are used to measure the total response of a material, whereas differential probes are used to measure the difference in the response between two coils. Encircling probes are used to inspect tubular components, whereas array probes consist of multiple coils that can be used to scan large areas. The choice of probe depends on the specific inspection requirements and nature of the material being tested [63,64,65].

Eddy currents are induced in the conductive regions of the composite, and the interaction of these currents with material properties can provide information regarding their condition. The electrical conductivity of CFRP materials depends on the volume fraction, structure, and orientation of the fibers, which can vary between the longitudinal and transverse directions. The conductivity of the fibers in the longitudinal direction is much higher (5000–50,000 S/m) than that in the transverse direction (10–100 S/m) [66].

Yi et al. [67] used eddy current pulse-compression thermography (ECPuCT) to characterize delamination in carbon CFRP materials. Compared with traditional single-pulse eddy current thermography, ECPuCT combines the Barker code-modulated eddy current excitation and pulse-compression techniques to improve the signal-to-noise ratio. The results show that the proposed ECPuCT approach can detect delamination depths ranging from 0.46 mm to 2.30 mm, and both the crossing point and skewness features have a monotonic relationship with delamination depth.

Pelkner et al. [68] conducted ECT on composite pressure vessels and found that it can detect leakage from both inside and outside on the CFRP vessel with a metallic liner. Moreover, ECT demonstrated considerable advantages in the detection of CFRP ropes. ECT offers a high inspection speed and the unique benefit of being a non-contact inspection method. It displays strong capability for detecting near-surface defects in CFRP ropes, making it a valuable technique for specific applications [56].

#### 2.2.2. Pulse Eddy Current Testing (PECT)

PECT is a time–domain electromagnetic NDT method developed to overcome the limitations of conventional sinusoidal ECT, particularly in the inspection of multilayer, coated, or corrosion-prone conductive materials. Unlike traditional ECT, which uses a continuous wave with a fixed frequency, PECT employs short broadband current pulses to generate transient eddy currents. These eddy currents diffuse over time, allowing the detection of both surface and subsurface flaws by analyzing the time-dependent response signal [69].

The operating principle of PECT is based on electromagnetic induction. The transmitter coil generates a pulsed magnetic field, inducing transient eddy currents in the conductive test object. These eddy currents diffuse and decay according to local conductivity, permeability, and the presence of discontinuities. A receiver coil captures the time-varying response, which is then analyzed to extract features such as the peak amplitude, rise time, decay rate, and zero-crossing time, which are indicative of the defect type, depth, and severity [69].

PECT offers several advantages over conventional ECT, notably, its enhanced penetration depth, reduced sensitivity to lift-off variations, and capability to operate through coatings or insulation. These features make it particularly valuable for corrosion under insulation (CUI) detection, aircraft fuselage inspections, and structural health monitoring (SHM) in offshore, aerospace, and automotive applications. For example, Zhang et al. [70] demonstrated a rectangular PECT probe for imaging and quantifying surface and subsurface metal losses in lightweight automotive alloy components. Their system utilized the peak amplitude and time–domain fitting of A-scan signals to accurately estimate the defect depth, which was further visualized using C-scan pseudo-3D imaging.

Sensor design is another area in which PECT has advanced significantly. Tytko et al. [71] proposed an innovative I-core sensor for the internal inspection of conductive tubes, addressing the limitations posed by external access, wall curvature, and lift-off variability. Their comparative analysis between air-core and I-core configurations showed that the latter achieved significantly higher sensitivity and impedance responses for both magnetic and non-magnetic materials, particularly when detecting inner-wall thinning and artificial defects.

Further enhancements in system accuracy and signal interpretation have been achieved using resonant eddy current sensors and digital signal acquisition methods. Ma et al. [72] introduced an LC resonator-based PECT system for inspecting CFRP materials. Their approach utilized inductance-to-digital converters (LDCs) to detect shifts in the resonant frequency, which correlate with changes in conductivity caused by defects. Their study confirmed an improved sensitivity and signal-to-noise ratio, even under variable lift-off and surface conditions.

PECT has also shown promise in the agricultural and environmental engineering contexts. Zhu et al. [73] analyzed the feasibility of using pulsed eddy current detection to assess soil salinization via conductivity changes. Their findings demonstrated that the depth of signal penetration and decay characteristics could be effectively correlated with soil conductivity, offering the potential for rapid and non-contact field assessment tools.

Hybrid systems have emerged in aerospace and marine applications, where defect access may be limited and coatings are prevalent. Zheng et al. [69] proposed a dual-mode composite probe that integrates PECT with electromagnetic ultrasonic testing (EMUT). Their experiments showed that although PECT is effective for surface and near-surface corrosion or thinning, EMUT complements it by detecting deeper flaws. This hybrid configuration improved the detection range and reliability of ship hulls, offshore platforms, and pipelines.

Despite these advancements, PECT is not without limitations. The skin effect and signal diffusion can limit the detectability of deep defects. The interpretation of time–domain signals, particularly under multilayered or anisotropic conditions, remains complex. Furthermore, sensor alignment, probe geometry, and environmental noise can introduce variability in the signal acquisition. To address this, researchers such as Zhang et al. explored digital filtering, linear fitting, and feature extraction techniques to improve the defect classification and localization [70].

Table 2 presents a comparative overview of electromagnetic-based NDT methods, specifically ECT and PECT. Although both techniques are grounded in the principle of electromagnetic induction, they differ in the signal excitation, detection mechanisms, and application scope. The table summarizes their key characteristics, including the operational principles, advantages, limitations, and typical industrial use cases, particularly for metallic and composite structures, such as CFRP. This structured comparison highlights the advancements offered by PECT over conventional ECT in terms of depth profiling and liftoff tolerance.

**Figure 2:**
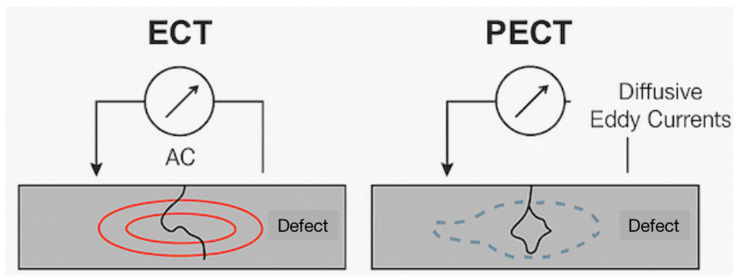
Schematic ECT and PECT operating principles.

Figure 2 provides a schematic representation of the operating principles of ECT and PECT. The ECT uses a sinusoidal alternating current to generate stable eddy currents near the surface, making it ideal for detecting surface-breaking flaws. In contrast, PECT employs pulsed excitation to induce transient diffusive eddy currents that penetrate deeper into a material. These differences are illustrated in the figure, emphasizing that PECT has enhanced capability for subsurface defect detection and inspection through coatings or insulation.

### 2.3. Acoustic Emission (AET)

AET is an NDT technique that involves the detection and analysis of elastic waves generated within a material owing to the release of energy from localized sources of damage or structural changes [74,75,76]. The detectable frequencies were in the range of 100 kHz to 1 MHz, far beyond the audible human range [77]. Within this frequency range, acoustic emission signals can be quantitatively analyzed using parameters such as the event count, event rate, signal energy, amplitude, and rise time [78,79,80]. The b-value analysis, which examines the statistical distribution of signal amplitudes, is particularly effective for identifying early stage damage accumulation and predicting imminent structural ruptures [81,82].

In the context of composite materials, AET can be used to identify and monitor various damage mechanisms, such as fiber breakage, matrix cracking, delamination, and debonding, which occur during the loading or service life of composite structures [83,84].

The AET has emerged as a valuable SHM technique for FRP composites. Guo et al. [85] demonstrated the effectiveness of the InceptionTime deep-learning approach for classifying different damage modes in composite materials using AET data. Acoustic events increase with increasing stress in composite materials and begin to accumulate as early as 10% of the ultimate failure stress [86].

Piezoelectric sensors including piezoelectric ceramics (PZT) and piezoelectric polymers (PVDF) are commonly used to detect AET signals in FRP composites. These sensors can be embedded within a laminated composite structure, creating a “smart material” that can continuously monitor the structural conditions [87]. Embedding the sensors directly within the composite offers advantages over the traditional NDT methods that require external inspection.

However, the incorporation of piezoelectric AET sensors into FRP composites presents several challenges. Factors such as the sensor size, placement, and coupling with the composite can influence the reliability and accuracy of AET measurements [88]. Researchers have also investigated methods to artificially induce AET events, such as the use of pencil lead breaks, to correlate AET signals with specific damage mechanisms in composites [89]. Table 3 lists the advantages and limitations of AET for composite materials.

**Figure 3:**
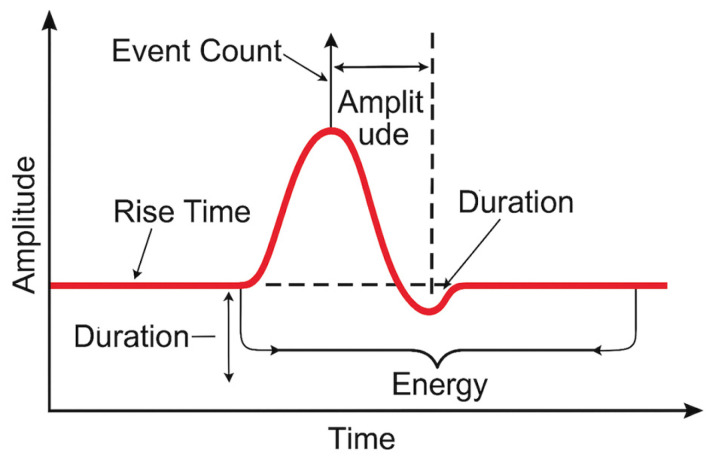
Signal parameters in acoustic emission testing.

Figure 3 illustrates the key signal parameters commonly used in AET for damage characterization. These include the rise time, amplitude, event count, duration, and energy, which are extracted from transient waveform signals generated during material deformation or cracking. Such parameters are critical for identifying damage mechanisms and predicting failure progression, particularly when used in combination with a b-value analysis.

### 2.4. Thermography (TR) and Infrared Thermography (IRT)

In the field of NDT, TR has emerged as a promising method for detecting and evaluating damage in composite materials. TR can be categorized into four main types: active thermography, passive thermography, transient thermography, and IRT. Table 4 lists the advantages, limitations, and real-world applications of the TR techniques.

IRT is a widely used TR technique that involves the use of an infrared camera to record the thermal radiation emitted by the specimen surface [90]. IRT does not require direct contact with a surface and can be applied to parts with complex geometries [91].

Zhang et al. [92] used IRT in combination with pulsed thermography (PRT) and vibrothermography (VRT) for defect detection in CFRP and glass-fiber reinforced polymer (GFRP) specimens. The effectiveness of these techniques was subsequently validated using PAUT. However, this type of inspection requires the use of sensitive and expensive instrumentation and highly skilled inspectors to operate the instruments. In addition, the clarity of the defects may be limited if they are located too deep within the part. Fang and Maldague [93] presented a method for estimating the depth of simulated defects in composite materials using IRT, demonstrating the potential of this technique for NDE.

The repeatability and reliability of IRT inspections are crucial for their widespread adoption in industry. Švantner et al. [94] presented a study on the repeatability of the flash-pulse thermographic inspection of CFRP samples, providing valuable insights into the consistency and reproducibility of this technique.

An experimental comparison of IRT, PAUT, and ECT in the detection of CFRP ropes yielded important insights. In the case of bare CFRP ropes, IRT proved to be effective in detecting damage and delamination. However, when the ropes were coated with a protective polymer, the IRT was limited because infrared radiation was blocked by the coating [56].

Chang et al. [95] explored the use of infrared imaging techniques to detect defects in composite materials, particularly those made of FRP. The authors discussed how IR technology can be leveraged to identify internal flaws, delamination, and other structural issues in composite components without causing damage [96].

Similarly, Li et al. [97] described a simulation approach for modeling the physical characteristics of IR imaging systems. This has enabled researchers to better understand the capabilities and limitations of IR-based NDT methods when they are applied to composite materials. Swiderski [98] focused specifically on the application of IRT for NDT of CFRP composites and demonstrated how this imaging technique can be used to detect various types of defects, including impact damage, delamination, and manufacturing flaws, within CFRP structures.

Active thermography is a technique that involves applying a periodic thermal stimulus to a material and analyzing the resulting thermal response. The active TR approach involves using an external heat source, such as halogen lamps, to thermally excite the component, causing it to emit infrared radiation that can be captured by an infrared camera [99].

Lock-in thermography is a type of active thermography that effectively provides information regarding the properties of a material and the presence of defects [100,101]. Similarly, Boccardi et al. [102] investigated the use of lock-in thermography and UT for the inspection of impact-damaged composite materials. This study highlighted the complementary nature of these two NDT techniques for evaluating the integrity of composite structures.

Peeters et al. [103] presented an optimized dynamic line-scan thermographic detection method for inspecting CFRP inserts. Researchers have used finite element (FE) modeling and probability of detection (POD) analysis to improve the performance of IRT techniques. Recent studies have focused on applying POD analysis to active thermography techniques, including pulsed thermography [104]. In one such study, a set of thirty-five CFRP composite panels with impact damage was inspected using both pulsed thermography and a UT C-scan. The comparative experimental results and POD analysis showed that pulsed thermography testing had a smaller defect size at 90% POD with a 95% confidence level (a90/95) than UT for the specific parameters and setup used in the inspections.

The development of robust and reliable NDT approaches is essential to ensure the structural integrity of composite structures in various industries, particularly in critical applications such as aerospace [105]. Laureti et al. [106] explored the application of pulse-compression thermography and air-coupled UT for the detection of delamination in polymeric ablative materials, which are commonly used in aerospace applications.

Passive thermography relies on the natural thermal response of the material. Summa and Herrmann [107] discussed the use of quantitative passive thermography to study the behavior of polymers. This technique can be particularly useful for monitoring the fatigue and degradation of composite structures over time.

Jiang et al. [108] explored the integration of simulations and IRT for the study of heritage materials, which can also be applicable to the investigation of composite structures. By combining experimental data with numerical simulations, researchers can gain a deeper understanding of the thermal behavior of composite materials and improve the reliability of damage detection.

Transient thermography involves the use of an external heat source to thermally excite a component, causing it to emit infrared radiation that can be captured by an infrared camera. Kim et al. [109] focused on the use of thermal science and engineering principles to develop numerical models and simulations for the detection of defects in composite materials. Such computational approaches can complement experimental techniques and help optimize the inspection and monitoring of composite structures. Wei et al. [110] presented a deep learning-based approach for the segmentation of impact damage in curved CFRP laminates using IRT. This study demonstrates the potential of combining artificial intelligence with TR techniques for the automated detection and characterization of defects in complex composite structures.

Liu et al. [111] investigated the use of pulsed thermography and lock-in thermography to enhance the detection of defects in these materials. In addition to defect detection, the evaluation of the mechanical behavior of composite materials under various loading conditions is an important area of research. Pirinu et al. [112] utilized IRT to study the response of composite materials to low-velocity impact events, which can lead to complex damage patterns that are challenging to characterize using traditional methods.

Another aspect of research on IRT and composites is the evaluation of the fracture behavior of these materials, particularly in terms of delamination [113]. Pitarresi et al. [114] employed IRT in combination with techniques such as thermoelastic stress analysis to investigate the static and fatigue-induced delamination behavior of FRP composites under mode II loading conditions. This is crucial for quality control and safety assessments in industries such as aerospace and automotive, where composite materials are increasingly used [115].

In addition to composites, IRT has also been explored for the detection and characterization of defects in other materials, such as titanium–graphite fiber metal laminates [116].

**Figure 4:**
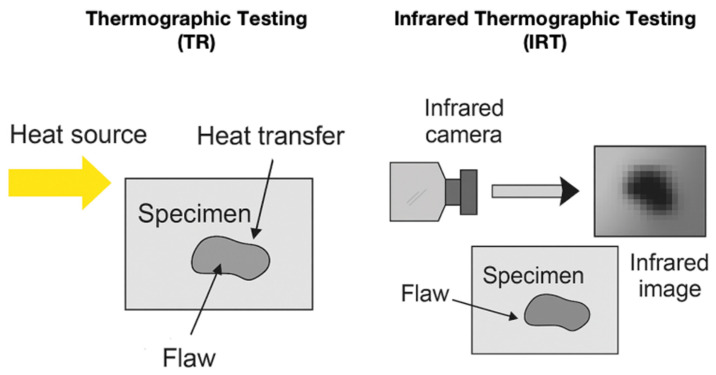
Schematic illustration comparing TR and IRT.

Figure 4 illustrates the working principles of the two thermographic-based NDT techniques, TR and IRT. Both methods rely on the thermal contrast generated by defects within materials; however, they differ in their mechanisms of heat delivery, detection approach, and analysis. In TR, an external heat source (such as a flash lamp or hot air) is applied to the specimen surface [117,118,119,120]. As heat propagates through the material, internal flaws such as delamination, voids, or cracks disrupt the uniform heat flow, which is then observed as thermal anomalies using infrared sensors [115,121,122,123,124,125]. In contrast, IRT emphasizes real-time infrared radiation detection using a thermal camera without requiring external heating, which makes IRT suitable for both passive (natural thermal gradients) and active (stimulated) inspection modes [92,126,127,128,129]. The infrared image generated reveals differences in the surface temperature distribution, which can be correlated to underlying material inconsistencies [130,131,132,133,134]. This figure highlights how each method visualizes internal flaws and demonstrates the growing applicability of IRT for evaluating composite structures with minimal surface preparation.

### 2.5. Microwave (MW)

MW technology, a type of electromagnetic radiation with wavelengths ranging from approximately one meter to one millimeter and frequencies between 300 MHz and 300 GHz [135], has been widely used in various applications, including communication, radar, and the NDT of materials. In recent years, MW has emerged as a promising approach for evaluating composite materials [136].

Researchers have investigated the use of free-space MW techniques to simulate and detect damages in CFRP composites [137]. The simulation results demonstrated the feasibility of the MW method for identifying damage in CFRP composites, thereby setting the stage for further experimental validation. Building on this foundation, researchers have examined the principles and applications of MW testing for both woven and nonwoven CFRP composites [138]. Their work provided a comprehensive overview of the successful application of MW to a range of CFRP composite materials, highlighting the versatility of this approach.

The potential of MW techniques has also been explored beyond that of carbon fiber composites. For instance, researchers have investigated the use of MW reflectometry for NDT of nonmetallic pipelines [139]. This exploration suggests the potential of MW techniques to be expanded to different composite-based infrastructure systems. Furthermore, the research community has studied the use of MW in FRP composites, indicating the broader applicability of MW methods across various composite-material systems.

Rahman et al. [2] employed MW on glass-fiber reinforced epoxy (GRE) and high-density polyethylene (HDPE) specimens containing holes and notches. They used one K-band circular waveguide probe and two (K- and K-band) rectangular waveguide probes and found that the circular waveguide probe showed better detection based on the GRE sample.

To improve the data analysis and interpretation capabilities of the MW techniques, researchers have developed sophisticated analytical approaches. One such effort involves the use of a k-means clustering algorithm to enhance the accuracy and reliability of MW for identifying defects in composite materials [140,141,142,143]. Table 5 lists the advantages and limitations of the MW for composite materials.

**Figure 5:**
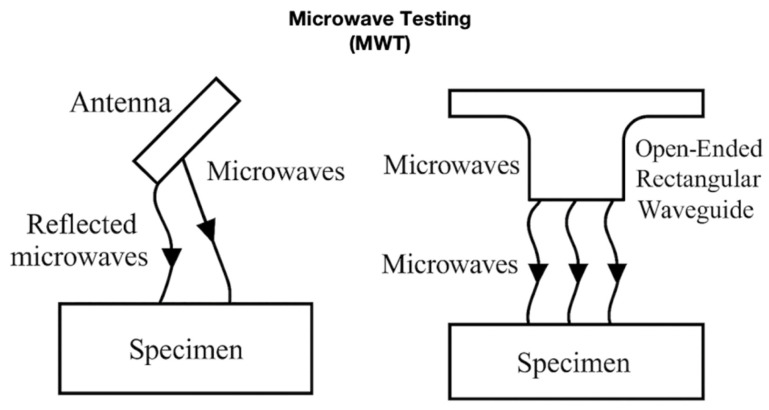
Schematic of microwave testing using an antenna and using an open-ended rectangular waveguide.

Figure 5 illustrates the two typical MW configurations used for composite and polymeric materials. In a free-space MW, an antenna directs microwave radiation toward the specimen, with reflected signals captured and analyzed for defect detection. In guided-wave setups, open-ended rectangular waveguides are used to improve the coupling efficiency and spatial resolution for localized flaw identification. Both configurations support various frequencies and probe geometries, such as K-band circular and rectangular waveguides, making them suitable for inspecting CFRP, GRE, and HDPE components.

### 2.6. Radiographic-Based Testing Methods

Radiographic-based NDT methods utilize ionizing radiation or X-rays to visualize internal structures and defects within composite materials. These techniques are particularly valuable for detecting voids, delamination, inclusions, and fiber misalignments, which may not be accessible to surface-based methods. Traditional RT relies on films or detectors to capture radiation-differential images, whereas modern DRT and XCT provide enhanced sensitivity, image processing capabilities, and 3D visualization. The following subsections describe these radiographic techniques in detail and highlight their respective capabilities and limitations.

#### 2.6.1. Radiography Testing (RT)

The RT is a widely used NDE technique that employs ionizing radiation to inspect and evaluate the internal structure and integrity of materials, components, and structures. The fundamental principle of RT is the differential attenuation of radiation as it passes through an inspected object, which is affected by the density and thickness of the material [144].

X-rays and gamma rays are the two primary forms of ionizing radiation used in RT applications [145]. Although both types of radiation can penetrate materials, there are key differences between them, as listed in Table 6.

The basic concept of RT applications involves placing the inspected object between the radiation source and detector, such as film or digital imaging sensors [146]. As radiation passes through an object, the varying densities and thicknesses of the material attenuate the radiation differently, creating a radiographic image that reveals the internal structure and any potential defects or anomalies [147].

RT is widely used in various industries, including aerospace, manufacturing, oil and gas, and infrastructure, to assess the integrity of materials, detect flaws, and ensure the safety and performance of critical components [148]. However, it is important to note that the use of ionizing radiation in RT applications carries potential health risks and must be carefully managed [149]. Exposure to high levels of radiation can have harmful effects on the human body, such as increased risk of cancer and radiation sickness. Strict safety protocols, including the use of shielding, remote handling, and personal protective equipment, are essential to minimize radiation exposure and ensure the safety of workers and the general public.

The unique properties and complex structures of composite materials present challenges and opportunities for RT applications. Composite materials composed of two or more distinct materials often exhibit significant variations in density and composition within a single component [5]. This heterogeneity can pose difficulties for traditional radiographic techniques, because the differential attenuation of radiation may not always provide clear and interpretable images.

Anoshkin et al. [150] conducted experimental studies to investigate the radiographic signs of major types of defects commonly found in polymeric composite materials (PCMs), such as interlayer delamination, pores, and wrinkles. A blade straightener was used as an example to evaluate the effectiveness of the microfocus X-ray method in detecting and characterizing these defects. The results of this study demonstrated that the microfocus X-ray method can detect defects as small as 0.5 mm in size. This high sensitivity to small defects makes the microfocus X-ray method a valuable tool for the NDE of PCM components, enabling the early detection of defects that could compromise the structural integrity of the material. The findings of this study highlight the potential of the microfocus X-ray method for ensuring the quality and reliability of PCM components in various industries.

Sahoo et al. [151] conducted a study on the quantitative assessment of thermal liner delamination in large-size composite rocket boosters using X-rays. This study aimed to address the limitations of UT in quantitatively assessing large delaminations and the lack of detailed information provided by tap testing. Although tap testing can reveal the presence of humps or defects, it does not provide comprehensive information regarding delamination size and extent. The results of this study demonstrated that RT could reveal additional delamination areas beyond those detected by tap testing, highlighting the effectiveness of RT in providing detailed and accurate information regarding the size and location of delaminations in large composite structures. The use of RT in this context shows its potential as a valuable tool for the NDE of composite materials in the aerospace industry.

Endrizzi et al. [152] characterized the drop-weight impact damage in composite structures by combining X-ray and UT C-scans, particularly those caused by low-velocity impacts. Low-velocity impact damage can lead to barely visible damage modes, such as matrix cracking, delamination, and fiber breakage. The UT C-scan showed large delamination and additional damage along the fiber directions. X-ray imaging was able to detect damage along the fiber directions, as well as other small-scale defects. X-ray imaging provides three sample representations: absorption, differential phase, and dark-field. Dark-field imaging is particularly useful for detecting cracks and voids smaller than the spatial resolution. However, the delamination orientation must not be parallel to the radiation beam [153].

#### 2.6.2. Digital Radiography Testing (DRT)

DRT is an RT technique that utilizes digital detectors instead of traditional film to capture radiographic images. The detector is connected to a computer that processes and displays the images on a monitor. DRT is widely used in various industries, including aerospace, automotive, manufacturing, and healthcare, to inspect and evaluate the internal structure and integrity of materials, components, and structures.

DRT offers several advantages over traditional film-based radiography, particularly in the inspection of composite materials. The high sensitivity and resolution of digital detectors enable the detection of small defects, such as delamination, cracks, and voids, which may not be visible using traditional methods. Furthermore, digital image processing techniques such as contrast enhancement and edge detection can improve the visibility of defects and facilitate image interpretation.

The use of DRT is particularly beneficial for inspecting complex geometries and thick composite materials, which can be challenging when using traditional RT techniques. The ability to acquire images quickly and process them in real time can also improve the inspection efficiency and reduce the overall cost of NDE. For instance, Li et al. [154] conducted case studies and field applications using DRT to inspect insulated pipelines, demonstrating its effectiveness in detecting defects without the need to remove insulation layers.

However, some challenges are associated with the use of DRT. Kusk et al. [155] investigated the impact of the anode heel effect on image quality in DRT. The anode heel effect refers to the non-uniform intensity distribution of the X-ray beam due to the angled orientation of the X-ray anode. This effect can lead to image artifacts and reduced image quality.

Kraai [156] discussed the advantages of digital detector array (DDA) technology over conventional film-based radiography, such as improved POD and faster throughput. However, DDA systems are more expensive than traditional film-based equipment, which presents an investment challenge for some industrial facilities. In addition, specialized training may be required to transition radiographic technicians from film-based techniques to digital techniques.

**Figure 6:**
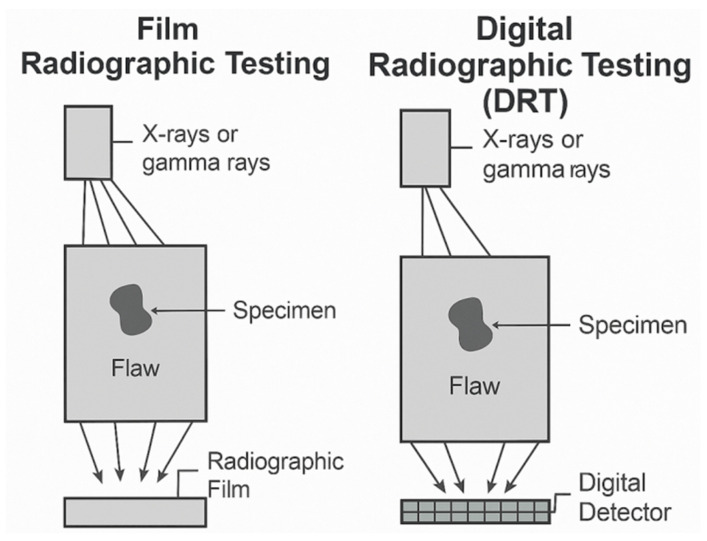
Schematic of radiographic testing using film and using digital detector.

Figure 6 presents a comparative schematic of RT and DRT, both of which rely on the attenuation of X-rays or gamma-rays as they pass through the specimen. In conventional film-based RT, a radiographic film placed behind an object captures the transmitted radiation, producing a static image that requires chemical development. In contrast, DRT replaces the film with a digital detector, such as a flat panel or imaging plate, allowing real-time acquisition, digital enhancement, and efficient data storage. This figure highlights the shared principles and distinct advantages of DRT in terms of speed, safety, and image-processing capabilities.

#### 2.6.3. X-Ray Tomography (XCT)

XCT has revolutionized the field of composite material research as a powerful non-destructive 3D imaging technique. By capturing a series of X-ray projection images at various angles and computationally reconstructing them into a 3D digital representation, XCT provides unparalleled insights into the internal features of complex materials. This information is vital to understand the relationship between the microstructure of a composite and its macroscopic, mechanical, thermal, and other functional properties.

Over the past decade, advancements in XCT technology have significantly expanded its applications in composite materials research. Improvements in spatial resolution, reduced acquisition times, and increased accessibility of laboratory XCT systems have enabled researchers to explore new frontiers in composite-material analysis.

A key application of XCT in composite material research is the investigation of holes drilled into CFRP composites [157]. By analyzing the quality and characteristics of these holes, researchers can optimize the assembly and joining processes for composite structures. XCT has also been instrumental in examining the internal damage and defects within sandwich composite structures subjected to low-velocity impact [158]. The insights gained from these studies provide valuable insights into the failure mechanisms of composite materials.

Moreover, XCT data have been used to develop predictive models for the mechanical properties of composite materials [159]. These models aid in the design and optimization of composite components, reducing the need for time-consuming and costly experimental trials. Additionally, synthetic datasets generated from XCT data have been employed to create automated segmentation algorithms and streamline the analysis of composite materials [160].

XCT has also provided an in-depth overview of the technical aspects and applications of polymer composites [161]. Researchers have used XCT to study the manufacturing processes, tensile and compression loading, fatigue, and impact damage to composite materials. Furthermore, methodical parametric studies have been conducted using XCT to determine the optimal image segmentation thresholds for accurately quantifying important microstructural features in composites, such as the fiber orientation and porosity [162].

The non-destructive nature of XCT allows for the evaluation of internal structures and damage states in “active twist” composite structures, which are designed to undergo controlled deformation for applications such as morphing aircraft wings [163]. In addition, XCT was compared with traditional destructive cross-sectioning and optical microscopy techniques, demonstrating that XCT can provide similar or superior information without sample preparation.

Multiscale XCT has been employed to assess the internal structure and impact behavior of ballistic-resistant composite panels made from thermoplastic polymer matrices reinforced with aramid fabrics [164]. XCT has also been used to investigate the damage initiation and progression in composite materials under loading [165]. To further enhance the capabilities of XCT, researchers have studied the effects of this technique on the properties and performance of composite materials [166].

Advanced image analysis techniques have been developed for the high-precision segmentation of challenging XCT data of composite samples, enabling accurate quantitative analysis [167]. Moreover, XCT has been combined with other NDT methods such as UT to provide a more comprehensive understanding of impact damage in composite structures [168]. High-resolution synchrotron radiation XCT (SR-XCT) has been utilized to investigate the microstructural evolution of composite materials during dynamic processes, such as oxidation at high temperatures [169].

**Figure 7:**
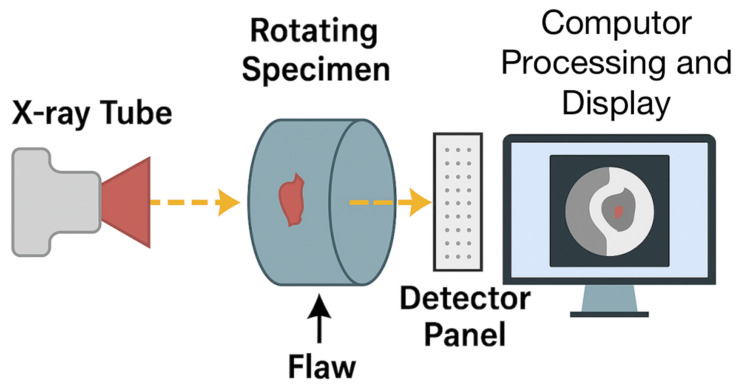
Schematic illustration of X-ray computed tomography (XCT).

Figure 7 illustrates the operating principle of XCT, which is a powerful NDT method that reconstructs 3D images of a specimen’s internal structure. In XCT, a specimen is placed between a rotating X-ray source and detector array. As the sample rotates, multiple two-dimensional (2D) radiographic projections are captured from different angles. These projections are then processed using tomographic reconstruction algorithms to generate a 3D volumetric image. This technique allows for the precise characterization of internal features, such as the porosity, delamination, fiber orientation, and embedded defects in composite and metallic materials.

Table 7 presents a comparative overview of radiographic-based NDT methods, including traditional RT, DRT, and XCT. Each method is described in terms of its working principles, advantages, limitations, and typical applications in composite-material inspection. This consolidated view highlights the evolution of conventional film-based techniques to advanced 3D imaging and digital diagnostics, providing insight into their suitability across different industrial contexts.

### 2.7. Comparative Classification of NDT Techniques

NDT techniques differ significantly in their physical principles, operating conditions, and defect-detection capabilities. This section provides a comparative classification of the previously described methods to facilitate a systematic understanding and address reviewer feedback. The key parameters considered include the operating frequency range of the technique, the nature of coupling or contact required, the types of defects that can be detected (e.g., cracks, delamination, voids), and the typical defect size resolution.

These factors are critical in selecting the appropriate NDT method for specific composite or metallic applications, particularly in the aerospace, automotive, and civil infrastructure sectors. For instance, while conventional UT offers deep penetration for internal defect detection, eddy-current-based methods such as ECT and PECT provide high sensitivity to surface and near-surface flaws. AET stands out for its capability for real-time damage monitoring, whereas radiographic and thermographic techniques can be used to visualize internal structures non-invasively.

Table 8 presents a unified comparison of all the electromagnetic, acoustic, thermal, and radiographic NDT techniques discussed in Section 2.1, Section 2.2, Section 2.3, Section 2.4, Section 2.5 and Section 2.6. This summary enables readers to quickly identify the most suitable method based on the inspection depth, defect size, and material condition.

## 3. Literature Review Summary and Key Findings

This section presents the results of a systematic review of 120 articles published between 2015 and 2025, with the aim of identifying the most studied and applied NDT methods for composite materials. This review shows clear research priorities, emerging innovations, and persistent gaps in the current body of knowledge.

### 3.1. Dominance of Ultrasonic and Thermographic Methods

The analysis confirmed that UT remains the most extensively researched and applied NDT technique for composite materials, accounting for approximately 45% of reviewed articles. Its widespread use is attributed to its capability to detect subsurface defects, such as delamination, disbonding, and porosity with reasonable reliability [6,41,42,43,44]. Despite this, UT faces limitations owing to the anisotropic nature of composite materials, which affects wave propagation, signal attenuation, and defect resolution. Advanced techniques, such as PAUT have been introduced to overcome these challenges, providing improved sensitivity, imaging flexibility, and faster scanning capabilities [41,42,43,44]. PAUT was reported in 20% of the reviewed studies, emphasizing its emerging role as a superior alternative to conventional UT for complex composite structures.

IRT, both passive and active, has been highlighted in 25% of the reviewed literature as a preferred method for non-contact surface and near-surface inspection [92,93]. IRT’s advantages include its applicability to large areas and complex geometries without requiring surface contact or couplants. However, environmental factors, such as the ambient temperature and surface emissivity, can affect its reliability, particularly for detecting subsurface defects deeper within the material [94,95,96].

### 3.2. Emerging but Underutilized Methods

Although traditional techniques dominate, this review also identifies promising emerging methods that remain underutilized in industrial applications. Notably, despite its non-contact nature, deep penetration capability, and environmental safety, MW testing has only been reported in 5% of the reviewed articles. The complexity of probe design and signal interpretation has limited its widespread adoption, although studies have highlighted its potential for detecting hidden defects in FRP and carbon CFRP composites [136,137,138].

Similarly, DRT and XCT have demonstrated exceptional imaging capabilities for internal defect visualization, including porosity, fiber misalignment, and delamination [150,151,152]. Despite this, concerns over radiation exposure, equipment cost, and operational safety limit their frequent use in routine inspections, particularly outside specialized industries, such as aerospace.

AET, appearing in 10% of the reviewed literature, presents an attractive solution for real-time SHM by detecting damage mechanisms, such as fiber breakage and matrix cracking during operation [85,87]. However, its effectiveness is limited by noise sensitivity and difficulty localizing acoustic sources in large or complex structures [88,89].

**Figure 8:**
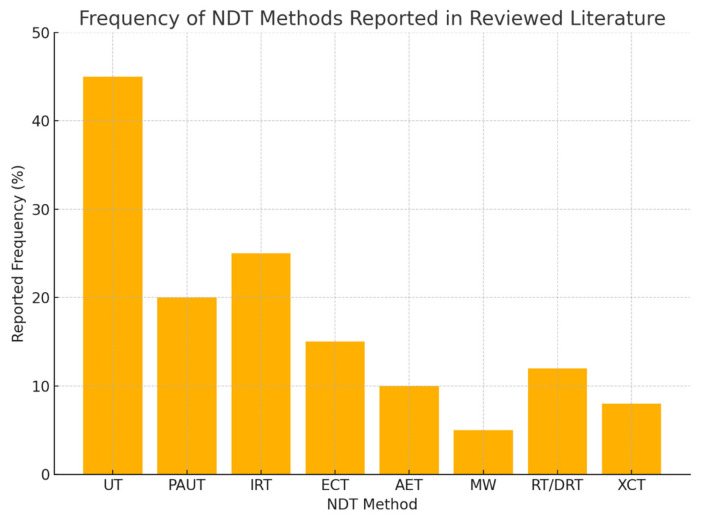
Reported frequency of NDT methods applied to composite materials based on the review of 120 research articles [1,2,3,4,5,6,7,8,9,10,11,12,13,14,15,16,17,18,19,20,21,30,31,33,34,35,36,37,38,39,40,41,42,43,44,45,46,47,48,49,50,51,52,53,56,57,58,64,65,66,67,68,72,74,75,77,81,83,84,85,86,87,88,89,90,92,93,94,95,97,98,99,100,101,102,103,104,106,107,110,111,112,113,114,115,116,122,123,124,125,126,128,129,131,133,135,136,137,138,139,140,142,149,150,151,152,153,157,158,159,161,162,163,164,166,168,169,170,171,172].

To complement the tabulated findings, Figure 8 presents a visual summary of the reported frequencies of each NDT method identified in the reviewed literature. UT and IRT clearly dominate, with emerging methods, such as MW and XCT, showing lower but growing research attention. This distribution highlights the reliance on traditional methods, while pointing to opportunities for diversifying industrial practices through advanced techniques.

### 3.3. Summary of Reported Frequency and Key Insights

Table 9 summarizes the frequency of NDT techniques reported in the reviewed literature and highlights their key findings. This comparative analysis not only reinforces the dominance of UT and IRT but also draws attention to underexplored yet promising methods such as MW and DRT.

This summary reinforces that while industry tends to favor UT and IRT for their reliability and cost-effectiveness, future research and application development should aim to expand the industrial readiness of advanced techniques, such as MW, DRT, and XCT, especially as manufacturing moves toward Industry 4.0 integration.

## 4. Perspective on Future Trends and Potential Applications

Based on the synthesis of 120 reviewed studies, it is evident that the NDT of composite materials has evolved significantly but still faces limitations that hinder its full industrial potential. As industries such as aerospace, renewable energy, oil and gas, and transportation increasingly rely on advanced composites, future research and industrial efforts must focus on overcoming these barriers by advancing the NDT capabilities in the following key areas.

**Figure 9:**
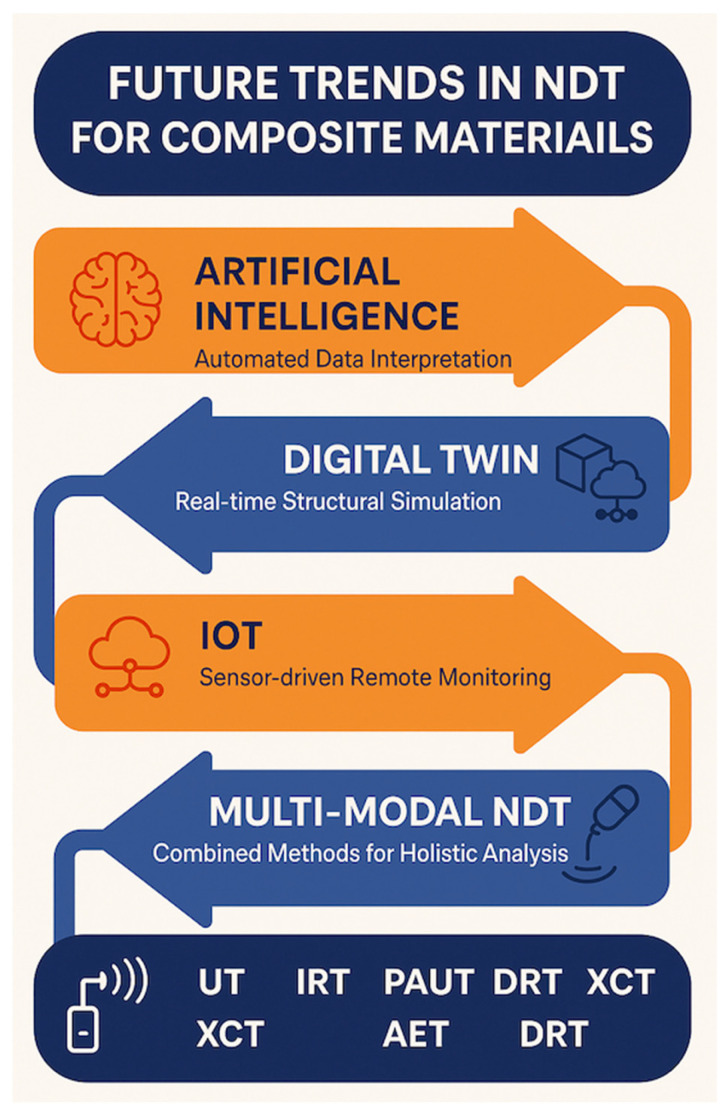
Technology roadmap illustrating four major future trends.

To frame the direction of future research and industrial implementation, Figure 9 presents a technology roadmap highlighting four interconnected trends poised to shape the next generation of NDT for composite materials. These include AI for automated data interpretation, digital twin technologies for real-time structural simulation, IoT for sensor-driven remote monitoring, and multimodal NDT for holistic defect detection through combined methods. These trends are expected to converge, transforming the traditional NDT into an intelligent, predictive, and fully integrated quality assurance system.

### 4.1. Adoption of Multimodal and Hybrid NDT Systems

One of the most significant advancements anticipated in the field of NDT for composite materials is the transition from single-method inspection to multimodal and hybrid systems. While individual methods such as UT, PAUT, IRT, and MW testing have demonstrated distinct advantages, their standalone application often limits comprehensive defect characterization, particularly in thick, multilayered, or anisotropic composite structures [41,42,43,44].

Multimodal NDT systems aim to overcome these limitations by integrating multiple inspection techniques into a unified framework, allowing complementary methods to work in parallel or in sequence. Such systems enable cross-validation of defect indications, improved sensitivity across various defect types, and enhanced localization accuracy. For example, although UT and PAUT provide high-resolution subsurface defect detection, IRT offers rapid, non-contact surface scanning, and MW testing contributes to deep penetration capability without requiring coupling agents.

**Figure 10:**
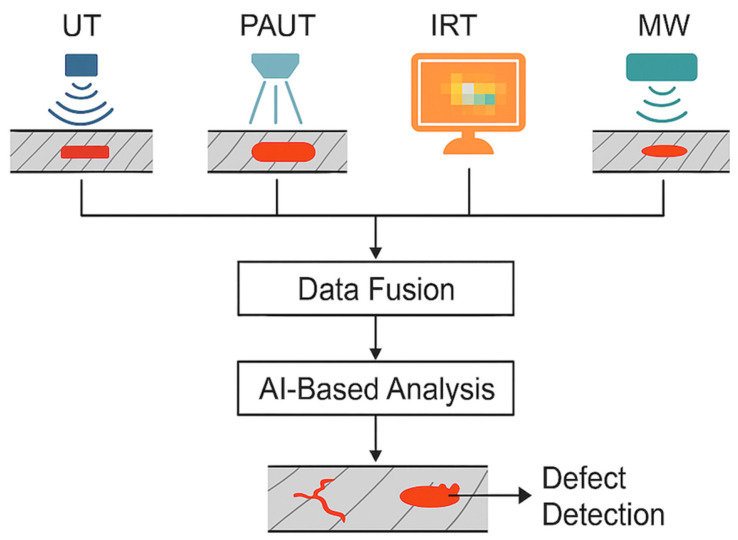
Schematic of a multimodal NDT system integrating UT, PAUT, IRT, and MW methods.

Figure 10 illustrates a conceptual schematic of a multimodal NDT system integrating the UT, PAUT, IRT, and MW methods. Each method is fed into a centralized data fusion module, where heterogeneous data are combined to create a more complete diagnostic profile [170,171,172,173,174]. These fused data are then processed through AI-based analysis, leveraging machine learning (ML) algorithms to automate defect classification and severity assessments [175,176]. The output is a detailed defect map that enables more accurate and reliable decision making for quality assurance and SHM [177].

### 4.2. Digital Twin and Industry 4.0 Integration

The next frontier in NDT is digitalization and real-time data integration. Digital twins, which are virtual models that replicate the physical behavior of composite structures, are gaining traction as predictive maintenance tools [178,179,180]. By integrating live data from NDT systems, such as AET and PAUT, these digital twins can simulate defect progression and estimate remaining useful life (RUL) [48,181,182]. Such predictive capabilities enable industries to transition from reactive to proactive maintenance strategies, reducing downtime, and extending asset life cycles [183,184,185,186].

### 4.3. Embedded and Wireless Structural Health Monitoring

Recent developments in embedded sensor technologies, such as PZT sensors, fiber-optic sensors, and wireless AET arrays, offer the potential for continuous in situ health monitoring without interrupting operations [187,188,189]. These embedded SHM systems allow for the real-time detection of damage mechanisms, such as delamination, fiber breakage, and matrix cracking [87,88,190,191]. The deployment of wireless and battery-free sensors further enhances the feasibility of integrating SHM into large-scale structures such as wind turbine blades, pipelines, and aerospace components [192,193,194,195,196].

### 4.4. Artificial Intelligence and Automated Defect Interpretation

As the volume of NDT data has increased with advanced sensing technologies, AI and ML offer powerful tools for automating data analysis. AI algorithms, including deep learning frameworks such as InceptionTime, have already shown promise for classifying damage types based on AET signals [85,197]. Future AI-driven systems will enhance defect characterization, reduce operator dependency, and enable faster decision making in manufacturing and maintenance workflows [198,199,200].

### 4.5. Environmentally Friendly and Operator-Safe Techniques

Although radiographic methods such as DRT and XCT provide high-resolution imaging, health and environmental safety concerns limit their routine use. Safer alternatives such as MW and ECT are gaining attention because of their non-ionizing nature, environmental safety, and effectiveness in detecting deep or hidden defects [97,98,99]. Promoting these safer NDT methods aligns with global occupational safety and sustainability goals.

To better illustrate the current technological landscape, Table 10 summarizes the key advantages and limitations of the major NDT techniques reviewed. This comparison not only highlights their strengths but also exposes critical gaps that justify the need for hybrid approaches, digital enhancements, and standardized implementation.

### 4.6. Standardization and Industrial Certification

Despite the technological advancements in NDT, the lack of standardized calibration procedures, reference materials, and data interpretation guidelines remains a significant barrier to its widespread adoption. The development of internationally recognized standards and certification frameworks is crucial to ensure repeatable, reliable, and comparable results across industries and applications [150,151,152,153]. Collaborative efforts among research institutions, industry leaders, and standardization bodies are essential to close this gap.

## 5. Conclusions

This review comprehensively analyzes the state-of-the-art NDT techniques applied to composite materials, including UT, PAUT, AET, IRT, ECT, MW methods, RT, DRT, and XCT. A systematic review of 120 research articles revealed that UT and IRT remain the most widely applied methods primarily because of their accessibility, reliability, and established industrial practices.

However, emerging methods such as MW, DRT, AET, and XCT, are still underutilized in practice. These techniques offer enhanced defect characterization, safer operation, and integration potential with digital manufacturing environments, but face challenges related to cost, data interpretation complexity, and a lack of standardized procedures.

The key findings of this review are as follows:

- No single NDT method is universally sufficient for all composite material challenges.
- Multimodal and hybrid NDT approaches offer the most comprehensive defect-detection capability.
- The integration of NDT with Industry 4.0 technologies, such as digital twins and real-time SHM, represents the next frontier.
- AI and ML have shown significant potential for automating defect recognition and improving data analysis reliability.
- Environmentally friendly and operator-safe alternatives such as MW and ECT should be further explored and promoted.
- A major gap exists in the standardization and certification of advanced NDT methods, which require collaborative efforts among industry, academia, and regulatory bodies.

### Future Outlook

To unlock the full potential of NDT in composite-material applications, future research and industrial efforts should focus on the following:

- Advancing hybrid inspection systems that combine the strengths of multiple methods.
- Developing AI-powered analytics for real-time defect interpretation.
- Promoting sustainable and safe inspection methods.
- Establishing internationally recognized standards and certification protocols to ensure consistency and industry-wide adoption.

In line with these directions, future research will explore the integration of sensor fusion and digital twin technology for real-time SHM. By combining advanced sensing hardware with virtual modeling environments, future NDT systems can move beyond passive defect detection to predictive diagnostics, enabling intelligent decision-making, risk-based maintenance, and extended asset lifecycles.

Addressing these future priorities will help elevate the NDT from a traditional quality control tool to a digitally enabled, intelligent infrastructure management platform tailored for next-generation composite structures.

## Figures and Tables

**Table 1:** Ultrasonic-based testing methods: description, advantages, limitations, and applications.

Method	Description	Advantages	Limitations	Typical Applications
Ultrasonic Testing (UT)	A conventional NDT method using high-frequency sound waves (typically 1–25 MHz) to detect internal flaws. A transducer sends pulses into the material, and reflections are analyzed to identify defects.	- Non-destructive and field-portable - Effective for internal flaw detection (e.g., delamination, voids, inclusions) - Measures thickness and material integrity - Cost-effective and widely available	- Signal attenuation in thick or anisotropic composites - Requires couplant (e.g., water or gel) - Less sensitive to small/tight flaws - Challenging for curved geometries	- FRP pipeline inspection - Aerospace structural panels - Wind turbine blades - Automotive composite parts
Phased Array Ultrasonic Testing (PAUT)	An advanced UT technique using multiple piezoelectric elements in a phased array to steer and focus ultrasonic beams. Enables dynamic beam control, sectorial scans, and improved resolution.	- Beam steering for complex geometries - Higher resolution than UT - Faster scanning and imaging - Can detect small, embedded defects	- Higher cost and complexity - Requires skilled operators and calibration - Sensitive to surface conditions - Depth penetration still limited in thick composites	- Thermoplastic composite pipe (TCP) inspection - Detection of fiber breakage and matrix cracks - Aircraft composite skin and rib inspections - Multilayer CFRP delamination detection

**Table 2:** Electromagnetic-based testing methods: description, advantages, limitations, and applications.

Method	Description	Advantages	Limitations	Typical Applications
Eddy Current Testing (ECT)	ECT uses alternating current to induce eddy currents in a conductive material. Variations in the induced currents—caused by flaws or changes in conductivity—are measured via electromagnetic coupling to detect surface anomalies.	- High sensitivity to small surface and near-surface defects - Fast and non-contact inspection - Suitable for metallic and CFRP materials - Works on complex geometries with appropriate probe selection	- Limited penetration depth (only effective for near-surface flaws) - Sensitive to lift-off and material properties - Less effective for non-conductive or highly anisotropic materials	- Crack detection in metallic pipes - Conductivity evaluation in CFRP panels - Surface corrosion monitoring - Quality control of heat exchanger tubes
Pulse Eddy Current Testing (PECT)	PECT uses short, broadband pulses to generate transient eddy currents. The decaying signal response is captured and analyzed in the time domain, allowing the detection of surface and subsurface flaws through coatings or insulation.	- Penetrates coatings and insulation - Effective for subsurface and multilayer defects - Less sensitive to lift-off than ECT - Enables defect depth profiling - Portable and suitable for field use	- Lower spatial resolution than conventional ECT - Signal interpretation can be complex and affected by noise - Requires specialized signal processing and calibration for layered or composite systems	- Corrosion under insulation (CUI) - Thickness loss in storage tanks and pipelines - Inspection of CFRP/GFRP laminates - Structural health monitoring in aerospace/marine

**Table 3:** Advantages and limitations of AET for composite materials.

Feature	Description	Advantages	Limitations	Typical Applications
Real-time damage detection	AET captures elastic waves released from localized failure sources within composite materials during mechanical loading.	- Enables early identification of failure modes - Monitors damage progression in real-time - Applicable for in-service SHM	- Requires continuous monitoring - Interpretation can be affected by background noise	- Aerospace composite panels - Pressure vessels - Structural fatigue testing
Signal parameters	Analyzes number of events, energy release, event rate, counts, amplitude, and frequency spectrum. b-value analysis is used to detect critical rupture precursors.	- Provides rich data for understanding damage mechanisms - Enables predictive failure modeling	- Requires advanced signal processing and calibration - Sensor placement critical for localization accuracy	- Fracture initiation studies - Fatigue crack growth monitoring
Smart structure integration	Embedded piezoelectric sensors (PZT, PVDF) enable continuous health monitoring from within the composite layers.	- No need for external inspection - Suitable for integration in next-gen SHM systems	- Sensor embedding may affect material integrity - Long-term sensor durability under stress is a concern	- Wind turbine blades - Aerospace composite spars - Marine sandwich panels

**Table 4:** Advantages, limitations, and real-world applications of thermography techniques.

Type of Thermography	Description	Advantage	Limitation	Real-World Application
Active Thermography	Pulsed Thermography: The object is briefly heated, and the cooling pattern is monitored to detect defects. Vibrothermography: The object is mechanically vibrated, causing heat generation at defect locations. Lock-in Thermography: The object is subjected to a periodic heating, and the phase and amplitude of the thermal response are analyzed.	Provides higher sensitivity and better defect detection compared to passive thermography. Can be used to actively excite the object and analyze the thermal response. Suitable for detecting subsurface defects and flaws.	Requires external heating or excitation sources, which can be complex and energy-intensive. The heating/excitation process can be time-consuming and may not be suitable for large or complex structures.	Inspection of composite materials and structures: Pulsed thermography has been used to detect delaminations, impact damage, and disbonds in CFRP composites. Nondestructive evaluation of aerospace components: Vibrothermography has been employed to detect fatigue cracks and other defects in aircraft parts and engines. Inspection of concrete and civil infrastructure: Lock-in thermography has been used to identify voids, delaminations, and other defects in concrete structures and bridges.
Passive Thermography	• Passive thermography relies on the natural thermal differences in the object, without applying any external heating or excitation. This is commonly used for condition monitoring and predictive maintenance applications.	Does not require external heating or excitation, making it a simpler and more cost-effective technique. Can be used for continuous monitoring and condition assessment. Suitable for large-scale structures and areas where the thermal differences are naturally present.	Relies on natural thermal variations, which may not always be sufficient for effective defect detection. Sensitivity is generally lower compared to active thermography. May not be able to detect subsurface defects as effectively as active methods.	Building energy audits and monitoring: Passive thermography is used to detect thermal bridges, air leaks, and insulation issues in buildings. Electrical equipment condition monitoring: Passive thermography is employed to identify hot spots and potential failures in electrical switchgear, transformers, and other power system components. Predictive maintenance in industrial settings: Passive thermography is used to monitor the condition of rotating machinery, bearings, and other industrial equipment to plan maintenance and prevent breakdowns.
Transient Thermography	• Transient thermography involves monitoring the surface temperature of an object as it cools down after a brief heating pulse. Defects are detected by analyzing the cooling patterns.	Provides information about the depth and size of subsurface defects by analyzing the cooling patterns. Can be used for a wide range of materials, including composites, metals, and concrete.	Requires precise control and timing of the heating pulse and thermal data acquisition. The depth of detection is limited by the thermal diffusion properties of the material.	Inspection of composite materials: Transient thermography has been successfully used to detect and characterize delaminations, impact damage, and porosity in CFRP composites. Evaluation of concrete structures: Transient thermography has been applied to detect voids, cracks, and delaminations in concrete bridges and buildings. Nondestructive testing of metal components: Transient thermography has been used to inspect welds, castings, and other metal parts for defects and irregularities.
Infrared Thermography (IRT)	• IRT is the most common type of thermography, which uses infrared cameras to capture the thermal patterns on the surface of an object.	Provides a non-contact, non-destructive way to capture and visualize thermal patterns on the surface of an object. Allows for real-time monitoring and inspection of large areas or structures. Can be used for a wide range of applications, from material characterization to condition monitoring.	The effectiveness of IRT can be influenced by environmental factors, such as ambient temperature, humidity, and air currents. Requires careful calibration and temperature compensation to obtain accurate thermal measurements. Subsurface defects may not be easily detected, depending on the material and defect characteristics.	Building energy efficiency and conservation: IRT is used to identify thermal bridges, air leaks, and insulation issues in buildings to improve energy efficiency. Electrical and mechanical equipment monitoring: IRT is employed to detect hot spots, overloaded circuits, and potential failures in electrical systems, HVAC equipment, and industrial machinery. Medical and biological applications: IRT is used for early detection of breast cancer, inflammation, and other medical conditions by analyzing the thermal patterns of the body.

**Table 5:** Microwave testing for composite materials: description, advantages, limitations, and applications.

Feature	Description	Advantages	Limitations	Typical Applications
Microwave Testing (MW)	MW-based NDT uses electromagnetic radiation in the 300 MHz to 300 GHz range to inspect dielectric or conductive materials. It measures reflected and transmitted waves to detect anomalies caused by defects or inhomogeneities.	- Non-contact and non-invasive - Environmentally safe (non-ionizing) - High penetration depth - Suitable for coated or multilayer composites - Supports advanced signal interpretation (e.g., S-parameters, AI integration)	- Requires complex antenna and probe design - Sensitive to surface roughness or coating interference - Lower spatial resolution compared to UT or XCT - Interpretation requires specialized signal processing	- Detection of delamination or moisture ingress in CFRP - Inspection of honeycomb sandwich structures - Nonmetallic pipeline defect detection - Aerospace and wind energy composite panels

**Table 6:** The different between X-ray and Gamma-ray.

X-Ray	Gamma-Ray
X-rays are produced by the interaction of high-energy electrons with a metal target, typically made of tungsten or copper, within an X-ray tube.	Gamma rays are emitted from the nucleus of radioactive isotopes, such as Iridium-192 or Cobalt-60, during the radioactive decay process.
The energy of X-rays can be controlled by adjusting the voltage and current applied to the X-ray tube.	The energy of gamma rays is determined by the specific radioactive isotope and cannot be easily adjusted.
X-rays have a wavelength range of approximately 0.01 to 10 nanometers, which is longer than the wavelength of gamma rays.	Gamma rays have a shorter wavelength and higher frequency than X-rays, typically ranging from 0.01 to 0.1 nanometers.
X-ray sources can be turned on and off, allowing for controlled exposure and better regulation of the radiation dose.	Gamma ray sources are continuously emitting radiation and cannot be turned off, requiring more stringent safety measures.

**Table 7:** Radiographic-based NDT methods: description, advantages, limitations, and applications.

Method	Description	Advantages	Limitations	Typical Applications
Radiographic Testing (RT)	RT uses X-rays or gamma rays to generate 2D images of internal features based on differential absorption in materials. Film or digital sensors capture the transmitted radiation to detect voids, inclusions, and delamination.	- Effective for identifying internal defects - Can inspect complex geometries - Compatible with both metallic and composite structures - Provides permanent image records	- Involves ionizing radiation (safety risk) - Limited depth penetration in thick composites - Image interpretation requires experience - Defect orientation affects visibility	- Aerospace component inspection - Thermal liner delamination in rocket boosters - Defect mapping in polymeric composites
Digital Radiographic Testing (DRT)	DRT is an advanced form of RT that replaces film with digital detectors, enabling real-time image acquisition, enhancement, and analysis with higher sensitivity and resolution.	- High-resolution imaging - Real-time inspection and faster processing - Enhanced image contrast and defect visibility - Reduces need for retakes	- Higher equipment cost- Requires digital image processing expertise - Image artifacts (e.g., anode heel effect) - Limited detector size for large components	- Insulated pipeline inspection - Weld quality assessment in CFRP and GFRP structures - Field inspection of infrastructure composites
X-ray Computed Tomography (XCT)	XCT reconstructs 3D internal structures from multiple 2D X-ray projections. It enables the precise visualization and measurement of internal defects like porosity, delamination, and fiber misalignment in composites.	- High-resolution 3D imaging - Enables quantification of fiber orientation, void volume, and porosity - Non-destructive and repeatable - Supports modeling and machine learning	- Long scan and processing times - Expensive hardware and high data storage needs - Limited sample size due to machine constraints - Requires advanced image segmentation skills	- Ballistic panel microstructure analysis - Drilled-hole quality inspection - Damage tracking in aerospace sandwich panels - Dataset generation for AI training

**Table 8:** Comparative classification of NDT techniques.

NDT Method	Operating Frequency	Coupling Requirement	Detectable Defects	Typical Defect Size Range	Applications
Ultrasonic Testing (UT)	0.5–15 MHz	Contact (gel)	Delamination, cracks, voids	0.1–1 mm	Composite panels, bonded joints
Phased Array Ultrasonic Testing (PAUT)	1–10 MHz	Contact (gel), automated scan	Delamination, cracks, porosity	0.1 mm or less	Aerospace laminates, CFRP
Eddy Current Testing (ECT)	100 kHz–10 MHz	Non-contact (lift-off sensitive)	Surface cracks, corrosion	0.1–1 mm	Conductive surface inspections
Pulsed Eddy Current Testing (PECT)	10 Hz–1 kHz (time–domain)	Non-contact (lift-off tolerant)	Subsurface corrosion, cracks	1–5 mm subsurface	CUI detection, corrosion mapping
Acoustic Emission Testing (AET)	100 kHz–1 MHz	Passive, no coupling	Crack initiation, delamination	Micron to mm (based on signal)	SHM, real-time monitoring
Infrared Thermography (IRT)	0.1–100 Hz (thermal)	Non-contact (IR camera)	Disbonding, surface defects	1–5 mm (thermal gradient)	Impact damage, debonding
Microwave Testing (MWT)	300 MHz–300 GHz	Non-contact (free-space/waveguide)	Delamination, internal voids	1–3 mm (CFRP)	CFRP, GRE, HDPE inspection
Radiographic Testing (RT)	Up to 10^18^ Hz (X-ray/gamma)	Non-contact (radiation exposure)	Voids, inclusions, delamination	50 µm–mm	Aerospace structures, composites
Digital Radiography Testing (DRT)	Up to 10^18^ Hz (X-ray)	Non-contact (digital detector)	Voids, inclusions, weld flaws	50 µm–mm	In-field CFRP/GFRP inspection
X-ray Computed Tomography (XCT)	Multiple projections (X-ray)	Non-contact, enclosed system	Porosity, fiber misalignment	<10 µm	3D microstructure analysis

**Table 9:** Key findings and frequency of use of different NDT techniques.

NDE Technique	Key Findings	Frequency of Use
Ultrasonic Testing (UT)	UT is widely used for its ability to detect internal defects such as delaminations, disbonds, and porosity. However, its effectiveness is limited by the anisotropic nature of composite materials, requiring advanced signal processing techniques.	45% of the reviewed articles utilized UT
Phased Array Ultrasonic Testing (PAUT)	PAUT overcomes some limitations of conventional UT by providing better defect detection and flexibility in complex composite structures. However, it requires specialized equipment and training.	20% of the reviewed articles employed PAUT.
Eddy Current Testing (ECT)	ECT is effective for detecting surface and subsurface defects but faces challenges in interpreting signals due to the complexity of composite materials.	15% of the reviewed articles discussed ECT.
Acoustic Emission (AET)	AET is valuable for real-time damage detection but is limited by environmental factors and the difficulty in localizing defect sources.	10% of the reviewed articles focused on AET.
Thermography (IRT)	IRT is a non-contact method suitable for various applications but is influenced by environmental factors and may not easily detect subsurface defects.	25% of the reviewed articles utilized IRT.
Microwave (MW)	MW offers high penetration depth and is environmentally safe but requires complex probe designs and advanced interpretation techniques.	5% of the reviewed articles discussed MW.
Radiography Testing (RT)	RT is effective in identifying a wide range of internal defects but carries potential health risks due to ionizing radiation.	20% of the reviewed articles employed RT.
Digital Radiography Testing (DRT)	DRT offers high sensitivity and resolution but is more expensive and requires specialized training.	15% of the reviewed articles utilized DRT.
X-Ray Tomography (XCT)	XCT provides unparalleled insight into the internal features of complex materials but is limited by data processing requirements and sample size restrictions.	10% of the reviewed articles discussed XCT.

Note: The total percentage exceeds 100% as some articles discuss multiple NDT techniques.

**Table 10:** Advantages and limitations of NDT techniques based on composite materials.

NDE Technique	Advantages	Limitations
Ultrasonic Testing (UT)	Cost-effective, effective for metal parts and assemblies, can detect internal defects.	Limited by anisotropic structure, high attenuation, and low signal-to-noise ratio of composites.
Phased Array Ultrasonic Testing (PAUT)	Overcome limitations of UT, capable of focusing and steer ultrasonic signals.	Requires specialized equipment and training.
Eddy Current Testing (ECT)	Quick and accurate inspection, can detect surface and subsurface defects.	Complexity of composite materials can pose challenges in the interpretation of ECT signals.
Acoustic Emission (AET)	Effective in detecting and identifying different damage mechanisms in real-time.	Limited by environmental factors and difficulty in accurately localizing the source of AET events in complex composite structures.
Thermography (IRT)	Non-contact, can provide a wide range of applications.	Effectiveness can be influenced by environmental factors, and subsurface defects may not be easily detected.
Microwave (MW)	High penetration depth, non-invasive, environmentally safe.	Complexity of probe design, interpretation of results can be challenging.
Radiography Testing (RT)	Can identify a wide range of internal defects within composite structures.	Use of ionizing radiation carries potential health risks and must be carefully managed.
Digital Radiography Testing (DRT)	High sensitivity and resolution, improved image quality, faster inspection times.	More expensive than traditional film-based radiography, specialized training may be required.
X-Ray Tomography (XCT)	Provides unparalleled insight into the internal features of complex materials, multi-scale analysis.	Data processing requirements, sample size restrictions, acquisition time can be limiting factors.

## Data Availability

No new data were created in this study.
